# The pulsating brain: A review of experimental and clinical studies of intracranial pulsatility

**DOI:** 10.1186/2045-8118-8-5

**Published:** 2011-01-18

**Authors:** Mark E Wagshul, Per K Eide, Joseph R Madsen

**Affiliations:** 1 Albert Einstein College of Medicine, Department of Radiology, Bronx, NY 10461, USA; 2 University of Oslo, Department of Neurosurgery, Oslo, Norway; 3 Harvard Medical School, Department of Neurosurgery, Boston, MA, 02115, USA

## Abstract

The maintenance of adequate blood flow to the brain is critical for normal brain function; cerebral blood flow, its regulation and the effect of alteration in this flow with disease have been studied extensively and are very well understood. This flow is not steady, however; the systolic increase in blood pressure over the cardiac cycle causes regular variations in blood flow into and throughout the brain that are synchronous with the heart beat. Because the brain is contained within the fixed skull, these pulsations in flow and pressure are in turn transferred into brain tissue and all of the fluids contained therein including cerebrospinal fluid. While intracranial pulsatility has not been a primary focus of the clinical community, considerable data have accrued over the last sixty years and new applications are emerging to this day. Investigators have found it a useful marker in certain diseases, particularly in hydrocephalus and traumatic brain injury where large changes in intracranial pressure and in the biomechanical properties of the brain can lead to significant changes in pressure and flow pulsatility. In this work, we review the history of intracranial pulsatility beginning with its discovery and early characterization, consider the specific technologies such as transcranial Doppler and phase contrast MRI used to assess various aspects of brain pulsations, and examine the experimental and clinical studies which have used pulsatility to better understand brain function in health and with disease.

## Introduction

Numerous homeostatic processes in the brain, such as cerebral blood flow and maintenance of interstitial fluid equilibrium, depend critically on the regulation of intracranial pressure (ICP) and fluid flow. While it is the mean pressure and flow which are most important in these processes, there are also systematic variations in pressure and flow which can play an important part in homeostasis. In the brain, the largest of these variations is due to the variation in blood pressure over the cardiac cycle, henceforth referred to as cardiac pulsatility. Other pulsatile variations, such as respiratory and vasomotor induced oscillations, do affect pressure and flow over time but have less of an effect compared to cardiac-induced variations. (Note: For the remainder of this review article, we will consider cardiac-induced pulsatility only, and refer to this simply as *pulsatility*). How changes in pulsatile pressure and flow in the brain might affect disease development and progression is a question of recent interest. In particular, in diseases such as hydrocephalus (HC) and traumatic brain injury (TBI) where changes in the biomechanical properties of the brain can lead to marked changes in pressure and flow dynamics, the role of pulsations is a potentially important one. In this article, we will review the study of cardiac-induced pulsatility over the last sixty years by looking at a) the key elements of the pulsatile waveform, b) measurement and analysis methods for pressure and flow pulsatility in the brain, c) an historical review of intracranial pulsatility and how it has led to an improved understanding of intracranial physiology, and finally, d) some speculation about where pulsatility research might take us in improving medical diagnosis and treatment.

### Pressure and flow "compartments"

**Figure 1:**
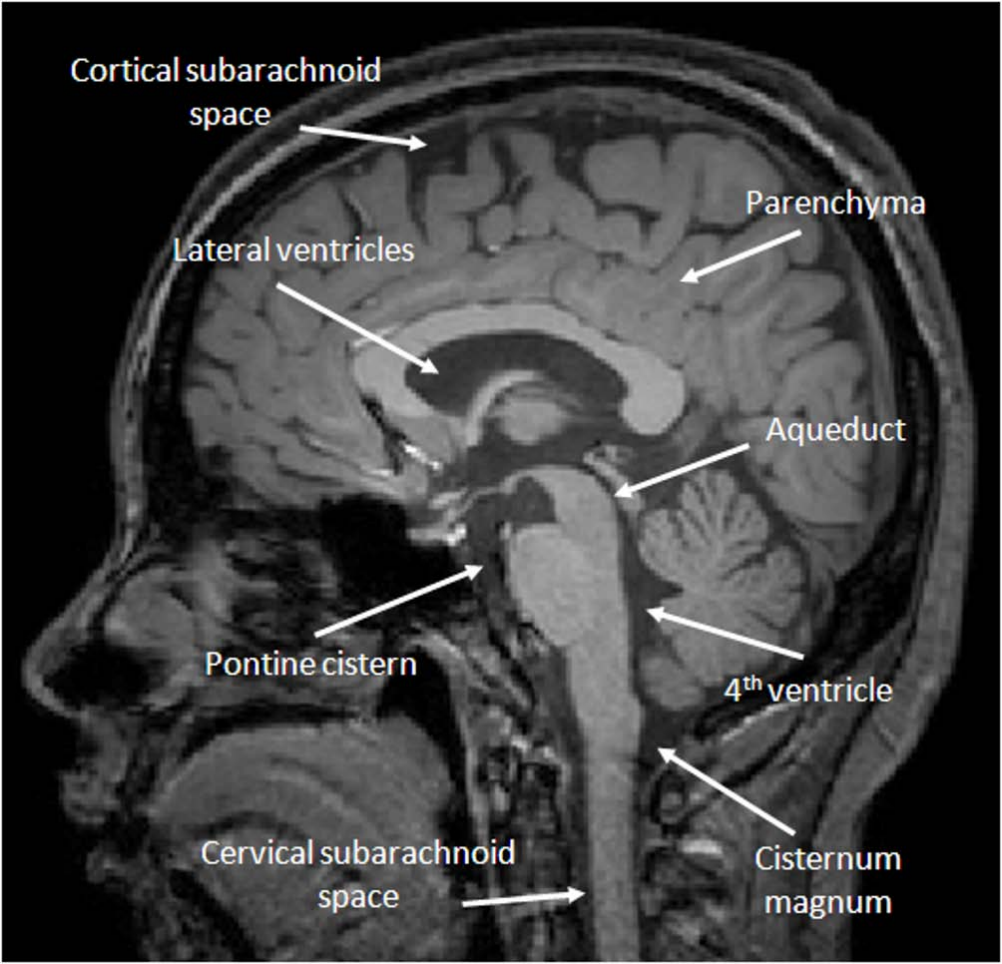
**Pressure and flow compartments in the brain**. Illustration of the pressure and flow "compartments" considered throughout the paper. Pressure can be measured anywhere within the cranium, and both mean pressure as well as pulse amplitude are generally considered to be position-independent. From a technical standpoint, however, pressure measurement is usually restricted to the lateral ventricles, cisternum magnum or the brain parenchyma. Flow, on the other hand, varies considerably with both magnitude (*i.e*., mean flow) and pulsatility strongly depending on fluid type (*e.g*., arterial blood vs. CSF) and on location. The figure indicates typical locations for CSF flow measurement. Blood velocity measurements (not shown) are generally restricted to the larger inlet/outlet vessels of the cranium (*e.g*., carotid, basilar, middle cerebral arteries, sagittal and straight sinuses).

The contractile variations in cardiac output have two distinct effects on intracranial dynamics, temporal changes in pressure and temporal changes in flow within the brain. While pressure and flow are related physical phenomena, they should be considered separately for one primary reason: pressure pulses propagate through the brain at the speed of sound and the exact point of measurement is usually not of great interest, while flow requires the displacement of fluid from one compartment to another and flow pulsations vary dramatically depending on location. Indeed, pressure can be measured almost anywhere in the brain and most studies of ICP dynamics have found that pressure pulsations in the brain are identical irrespective of location [1,2] (*e.g.*, whether measured in the ventricle, in the cisternum magnum or in the parenchyma). Flow pulsations throughout the brain, on the other hand, are highly dependent on the location chosen (*e.g*., from tens of centimeters per second within intracranial arteries to millimeters per second within the subarachnoid spaces). Figure 1 illustrates the relevant intracranial compartments considered. While there is certainly pulsatile flow within other compartments, such as interstitial fluid and the brain parenchyma itself, we will focus primarily on macroscopic fluid flow which is readily accessible with noninvasive measurement techniques.

### Pulsatility and compliance

**Figure 2:**
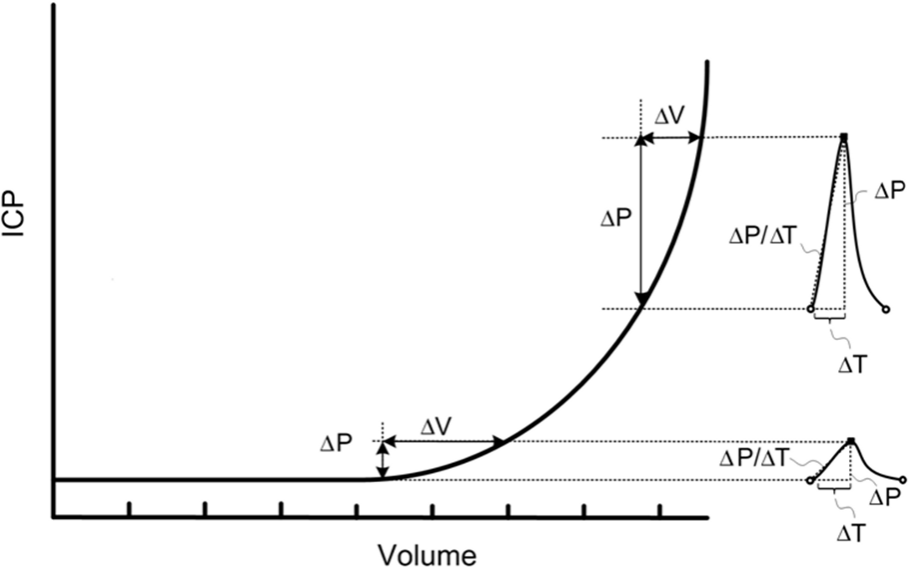
**The normal exponential pressure-volume relationship of the cranium**. The increase in pressure pulsatility with increased mean pressure is a result of the relationship between pressure and volume, which follows an exponential curve. At normal intracranial pressure (ICP) levels, the increase of intracranial blood volume in systole leads to a small increase in intracranial pressure, hence a normally small intracranial pulse wave (lower waveform, typical amplitude ~ 1 mmHg). With increases in intracranial pressure, the concurrent reduction in intracranial compliance leads to a dramatic increase in the pulse wave, even with no change in the arterial pressure wave (upper waveform). The intracranial pressure-volume curve was first introduced by Marmarou *et al *in 1975 [3], from which this figure was adapted.

It has been recognized for quite some time that pressure and flow pulsatility can change with disease; this has been used as a diagnostic tool in a number of areas. These changes are mostly due to the dependence of volume change on mean pressure, as first described by Marmarou *et al *for brain tissue [3], and based on the exponential pressure-volume relationship in the cranium (see Figure 2). It is important to understand that this exponential relationship is not a fundamental property of tissues, fluid, or flow, but rather a reasonable mathematical approximation based on observed data. It reflects the observation that a change in volume, such as during a systolic inflow of blood, is generally accompanied by a change in pressure, and that the magnitude of the change rises exponentially with mean pressure. The exponential relationship dictates that as the mean pressure increases, so do the pressure pulses (even though the volume of blood has not changed).

To fully understand the pressure-volume curve, it is important to introduce the concept of compliance (C), the ratio of volume (V) change to pressure (P) change, C = ΔV/ΔP. In a high compliance system, a large increase in volume will only result in a small increase in pressure. Conversely, in a low compliance system, only a small increase in volume can lead to a significant pressure rise. Graphically, compliance is the inverse of the slope of the pressure-volume curve. Thus, for the two pressure waves shown in Figure 2, the compliance is high in the lower waveform and low in the upper waveform. The exponential pressure-volume curve indicates that the compliance of the system is reduced simply because of the increased mean pressure. This makes sense intuitively. As the pressure rises, the system becomes more rigid and more sensitive to slight variations in volume.

Aside from the change in compliance with mean pressure, there are other potential sources of compliance change in the body which need to be considered, the most important being vascular compliance (*e.g*., hardening of the arteries with arteriosclerosis), which can affect pulsatility even in the absence of mean pressure changes. Thus, pulsatility can increase in a disease process involving either increased mean tissue pressure, or decreased tissue compliance. Examples of diseases exhibiting increased pulsatility abound: 1) age-related macular degeneration, in which intraocular pulsatility increases with disease severity [4], 2) peripheral vascular disease [5,6], 3) liver cirrhosis [7-9], and 4) dementia [10,11], to name a few. This list highlights the fact that pulsatility can be a valuable tool in disease assessment.

### The brain as a pulsatile organ

Most clinical applications of pulsatility have been outside of the brain, and the cranium presents a unique challenge for measuring pulsatility as well as a unique biomechanical environment for pulsatility. The predominant theory of non-steady blood flow in the human body is the Windkessel model, in which the elastic arterial walls serve as a storage mechanism for flow pulsatility, transforming pulsatile arterial blood flow into steady peripheral flow [12]. Because of the high compliance of the peripheral tissues, this mechanism is easily accomplished outside of the cranium, allowing the systolic arterial pulse wave to be transmitted and effectively dissipated in the surrounding tissue. The result is significantly attenuated microvascular and venous pulsations.

The brain in contrast is enclosed in a rigid container, and any transfer of pulsatility from the arterial walls into the surrounding tissue is felt almost instantaneously everywhere throughout the cranium. This leads to the observation noted above that intraparenchymal and CSF pressure waveforms tend to be similar and independent of location. This is sometimes over generalized to suggest that pressures are everywhere equal intracranially, but this obviously does not apply to the very important arterial and venous compartments. Secondly, this leads to the interesting and potentially important phenomenon of measurable flow pulsatility in the microvasculature [13] and in the venous system. In the brain, the substitute for tissue compliance, which dissipates arterial pulsations in non-cranial tissues, is the overall intracranial compliance. This compliance, is comprised of four main components: actual brain tissue compliance (which is small), arterial compliance, venous compliance (veins have highly compliant walls) and compliance of the spinal thecal sac (which communicates with the brain via the cerebrospinal fluid spaces). Traditionally, intracranial compliance is assumed to decrease primarily with increased ICP, due to the exponential pressure-volume relationship described above [14]. As was shown above, decreased compliance with elevated ICP leads to increased pressure pulsatility. However, an additional factor which must be considered is the transfer of pulsations out of the cranium through either venous or CSF outflow pathways; while usually not considered as a factor which affects intracranial compliance, this is another way in which pulsatility is modified in the brain. Thus, intracranial pulsatility can also be affected by restriction of these flow pathways (which can manifest itself as a change in either pressure or flow pulsatility), such as with venous hypertension or a blockage in the outflow CSF pathways at the craniocervical junction (*e.g*., in Chiari malformation or Dandy-Walker variant).

## How pulsatility is measured and key elements of the pulse wave

**Figure 3:**
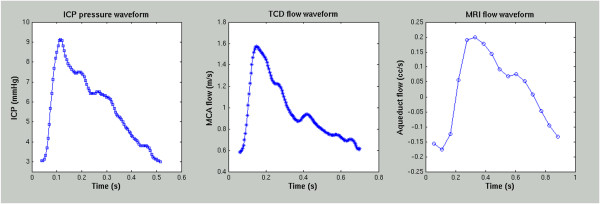
**Single pulse waves using the three primary methods reviewed in this paper**. Most noteworthy are the morphological differences between these waveforms, with the ICP pulse illustrating significant inter-pulse variations (known as P1, P2 and P3), mostly a result of pressure changes from the opening and closing of the cardiac valves, which are missing or attenuated in the middle cerebral artery blood flow waveform measured with transcranial Doppler ultrasound (middle panel), or in the aqueductal CSF flow waveform measured with phase contrast MRI (right panel). The marked reduction in temporal resolution with MRI as compared to ICP or TCD is also evident, and is due to the fact that MRI information is image-based and therefore much slower than single-point measurement techniques; the flow waveform data are acquired over many minutes and a single pulse wave is generated by averaging over many cardiac cycles.

Before proceeding to discuss the pre-clinical and clinical uses of pulsatility measures, it is important to understand the techniques for measuring pulsatility in the brain. Three primary techniques have been used to quantify aspects of intracranial pulsatility: continuous ICP monitoring, transcranial Doppler ultrasound (TCD), and magnetic resonance imaging (MRI). ICP monitoring, which is invasive, is used to measure pressure pulsatility and requires placement of a pressure sensor within the brain, either in parenchyma, ventricle, epidural space or the spinal CSF space. In comparison, TCD and MRI provide measures of flow pulsatility and have the distinct advantage of being non-invasive: TCD measures the velocity of blood flow in the large arteries using a transducer placed against the skull, while MRI measures the net flow waveform over the cardiac cycle, within the large intracranial arteries or veins or within well-defined CSF pathways (*e.g*., the cerebral aqueduct or at the craniocervical junction (CCJ)). Thus, ICP is a pressure-based measure of pulsatility, while TCD and MRI are flow-based. Accordingly, it is important to keep in mind that comparing pulsatility measures across modalities is not always valid, because the methods are not equivalent and assess different aspects of cardiac pulsatility. Examples of single cycle pulse waves using these three methods are illustrated in Figure 3.

### Intracranial pressure

Monitoring of ICP waves requires placement of a sensor within the skull (either in the brain parenchyma or within a ventricle), or in the spinal compartment. While this technique has been used by many investigators in pre-clinical work, there are only a few centers studying and using pressure pulsatility clinically. To some extent, this is due to the requirement of an invasive, implanted sensor, but it is also likely due to the difficulty of obtaining artifact-free pressure measurement in a clinical setting. As opposed to TCD and MRI, which are taken as one-time measurements with direct patient-operator interaction and good cooperation, ICP monitoring is typically done over a long time period with limited interaction between the patient and the operator. Thus, pressure signals are often corrupted by artifacts such as patient motion and heart rate variability. In addition, standard ICP monitoring software is only equipped to accurately measure mean ICP and is not easily accessed to extract the pressure waveform. Software for automatic identification of cardiac induced ICP waves and for dealing with artifacts has not been readily available (although the Sensometrics software package from dPCom has CE-mark for use in Europe). Thus, clinical examples in the literature using pressure pulsatility are limited.

**Figure 4:**
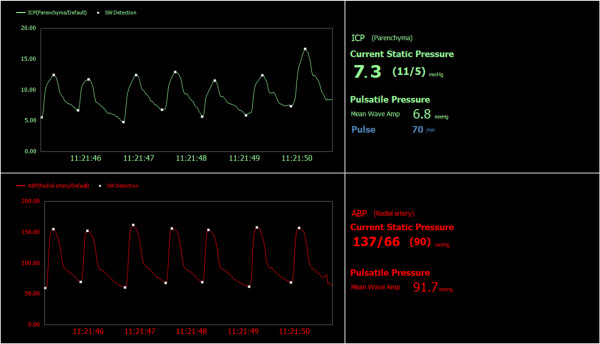
**Examples of pressure wave recordings**. An example of mean wave amplitude measurements taken from a clinical case at Oslo University Hospital, and showing simultaneous intracranial pressure (upper) and radial artery pressure (lower) waveforms. Automatic detection of pressure peaks and valleys allows for automated calculation of mean pressure, pulse amplitude (mean wave amplitude), and pulse latency (relative to the radial artery pulse pressure, 80 ms in this case).

The primary amplitude measure of pressure pulsatility is the absolute pulse amplitude, that is, the nadir-to-peak variation in pressure. This can be assessed either in the time domain, by measuring the nadir-to-peak (*i.e*., diastolic to systolic) amplitude of the pressure wave over one cardiac cycle, or in the frequency domain, by measuring the amplitude of the fundamental cardiac component (and possibly the first few harmonic components as well). Investigators have used the pulse pressure amplitude in the time domain as an indicator of intracranial compliance [15-20], and thus as a good indicator of HC severity and prognosis, but this never gained widespread clinical use, most likely due to the technical expertise required (*e.g*., accurately identifying cardiac-induced ICP waves) and the invasive nature of the procedure. Eide recently introduced a reliable and automated method for reliable identification of pulse pressure waves, incorporating three basic elements: (a) automatic identification of cardiac beat-induced pressure waves (in contrast to artifact-induced pressure waves), (b) characterization of individual pressure waves based on minimum diastolic pressure, maximum systolic pressure, pulse amplitude (*i.e*., diastolic-to-systolic pressure difference), rise time, and rise time coefficient (*i.e*., an approximation of dP/dT), (c) and presentation of the static pressure and pulse amplitude as a clinically useful output (see Figure 4). Averaging over a six-second time window, the pulsatility is represented by the mean wave amplitude. By monitoring this amplitude over a long period of time (*e.g*., many hours), a representative picture of pulsatility is obtained. Such measures have been used to show the importance of pressure pulsatility in diagnosis and shunt prediction for pediatric [21,22] and normal pressure hydrocephalus (NPH) [23-26], as well as for prognosis following TBI [27].

**Figure 5:**
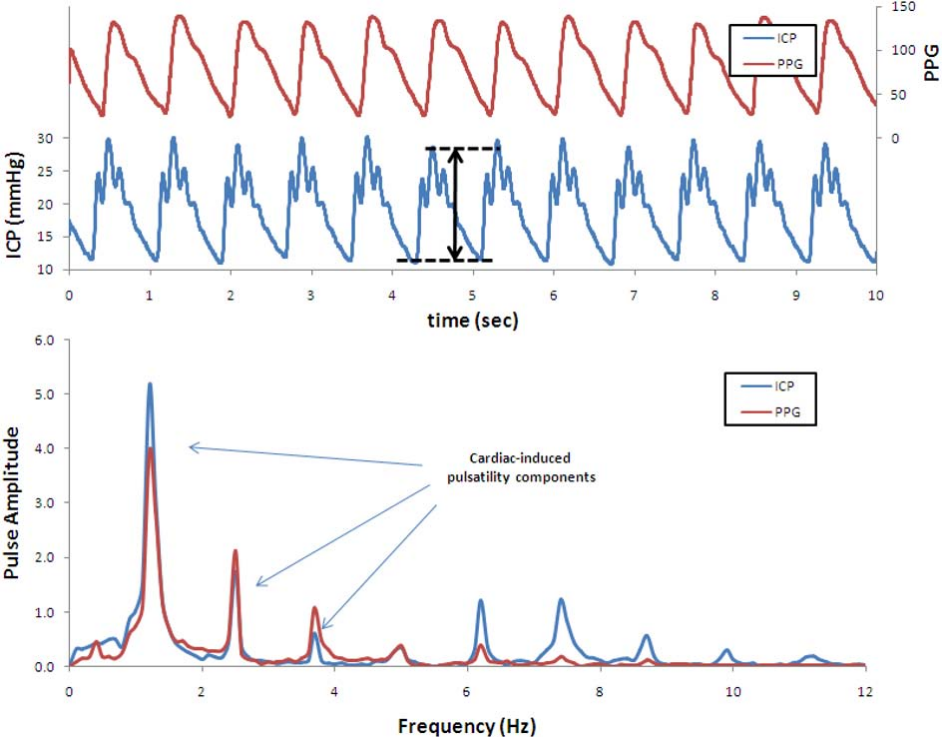
**Example of time- and frequency-domain pressure recordings**. In most clinical applications, data are presented, and analyzed, in the time domain (upper panel). In this case, the pressure is plotted as a function of time. In this example, the mean pressure (5.9 mmHg) as well as the pulse pressure (2.7 mmHg) can be extracted from the plot, although there can be confounding modulation of the pulse pressure from other sources such as respiration. Timing information can be extracted from the difference in timing of the peaks or troughs of the signal compared to the reference waveform (PPG, photoplethysmograph, in this case). In comparison, pressure data analyzed in the frequency domain is represented as a function of frequency (lower panel), and the signal now has well defined cardiac components which are easily separated from the low frequency components such as respiration and can be analyzed independently. Additional information available with frequency-domain analysis is the phase, the frequency-domain analog of timing in the time-domain (not shown). The phase plot allows analysis of timing differences between the ICP and the reference waveform for each identified frequency component.

An alternative method for determining pressure pulsatility is in the frequency domain, using the fast Fourier transform, which has been available since the late 1960's. After Fourier transformation, a pressure waveform is broken down into its individual frequency components, and the most prominent component is usually at the heart rate, due to cardiac-induced pulsatility. Portnoy and Chopp did extensive testing of pulse amplitude in the frequency domain, investigating changes in pulse amplitude in dogs with changes in physiology [28-30]. Czosnyka *et al *developed a method for determination of the amplitude of single pressure waves in the frequency domain. Using this approach, the frequency spectrum is determined using a small time window of the pressure waveform and the amplitude of the single waves is derived from the first harmonic component [31]. Figure 5 highlights the differences between time-domain and frequency domain measurements.

This approach has been implemented in software, which is used clinically (a commercial package, ICM+, is based on these methods [32]). The main advantage of the frequency domain approach is that it does not require identification of individual cardiac-induced waves. Although one group has shown measurable differences between time-domain and frequency-domain analyses [33], these might be improved by incorporating the higher harmonics in frequency-domain analysis. Indeed, a number of groups have used several harmonics of the spectrum to assess pulsatility [34-36], and shown promising results in TBI. Of course, frequency-domain techniques face the same technical dilemmas noted above, and have also not found widespread clinical acceptance.

### Transcranial Doppler ultrasound

TCD is used to non-invasively measure flow in the major arteries entering the brain, most commonly the middle cerebral artery, although other cerebral arteries are accessible [37,38]. The major advantages are that it is relatively inexpensive and quick to perform, and can be done successfully in most subjects with good cooperation. This technique provides two important measures: a) mean blood flow velocity, a relative measure of the integrity of arterial perfusion, and b) pulsatility index (PI) [39], a value reflecting cerebrovascular resistance and intracranial compliance. The main disadvantage of this technique is that insonation of the cerebral arteries of interest is not possible in a certain percentage of patients due to suboptimal insonation angle (10-20% of patients, [40]). The measure of blood flow is only a relative measure of perfusion integrity because velocities are measured, not absolute flow. Also, it is important to keep in mind that PI obtained with TCD is a measure of *vascular *velocity pulsatility, which is certainly related to the pressure pulsatility measures obtained with invasive pressure monitoring, but the relationship is not necessarily simple or linear.

The output of a TCD measurement is a velocity waveform as a function of time, for the entire recording period which is typically many cardiac cycles. This waveform can then be quantified in terms of the amplitude of the waveform, which is generally expressed as the PI, calculated as (peak systolic velocity - peak diastolic velocity)/mean velocity. Because it is normalized to the mean velocity, this is a measure of *relative *vascular pulsatility. A relative measure is used because of the difficulty of quantifying absolute velocity in a vessel; the velocity measured can vary dramatically depending on the size of the vessel, and the angle between the transducer and the vessel. PI, however, is insensitive to these experimental details and is a good gauge of changes in arterial pulsatility. One potential issue with PI measures, as compared to absolute pulsatility measures, is the dependence on both pulsatility and mean velocity; an increase in PI may not be strictly due to an increase in pulsatility but may also arise due to a decrease in mean velocity (*e.g*., decreased blood flow). Another measure frequently used clinically which is related to the PI is the resistive index (RI), defined as (peak systolic velocity - peak diastolic velocity)/peak systolic velocity. The advantage of RI is that it does not require integration of the flow parameter to determine mean velocity. RI has been associated with the probability of requiring a shunt in neonates with post-hemorrhagic hydrocephalus [41], although it is virtually certain that the PI would have made similar predictions in this setting.

### Magnetic resonance imaging

The technique of phase contrast MRI, in which quantitative velocity information is extracted from the MRI image, led to the non-invasive investigation of flow patterns in the brain [42]. Furthermore, by synchronizing the acquisition of the images to the cardiac cycle, it is possible to obtain velocity information as a function of the cardiac cycle - so-called cine phase contrast [43-46]. One important distinction of MRI, as compared to TCD and ICP, is that the MRI measurement is not taken in real-time; instead, the image must be collected over many cardiac cycles, so that the resultant velocity waveform is an average measure over many cycles. Thus, the MRI measure only generates a waveform consisting of one cardiac cycle (*i.e*., the waveform depicted in Figure 2 is the entire acquired dataset for MRI), while the real-time TCD and ICP measures generate waveform information over multiple cycles (*i.e*., the ICP and TCD waveforms depicted in Figure 2 are only a small fraction of the entire acquired dataset).

This new technology enables measurement of either CSF or blood flow pulsatility. While this technique is similar to TCD in providing absolute velocity information, it has the added MRI-specific advantage that image information is two or three-dimensional and net flow measurements can be extracted. Thus, quantitative measures of both velocity and flow pulsatility are obtained. Numerous vascular structures can be evaluated in a single image, with the only limitation being the size of the vessels (typically limited to vessels > 2 mm in diameter) [47]. By varying the velocity sensitivity of the technique, called the encoding velocity or V_enc_, CSF flow regions can also be assessed [48,49].

**Figure 6:**
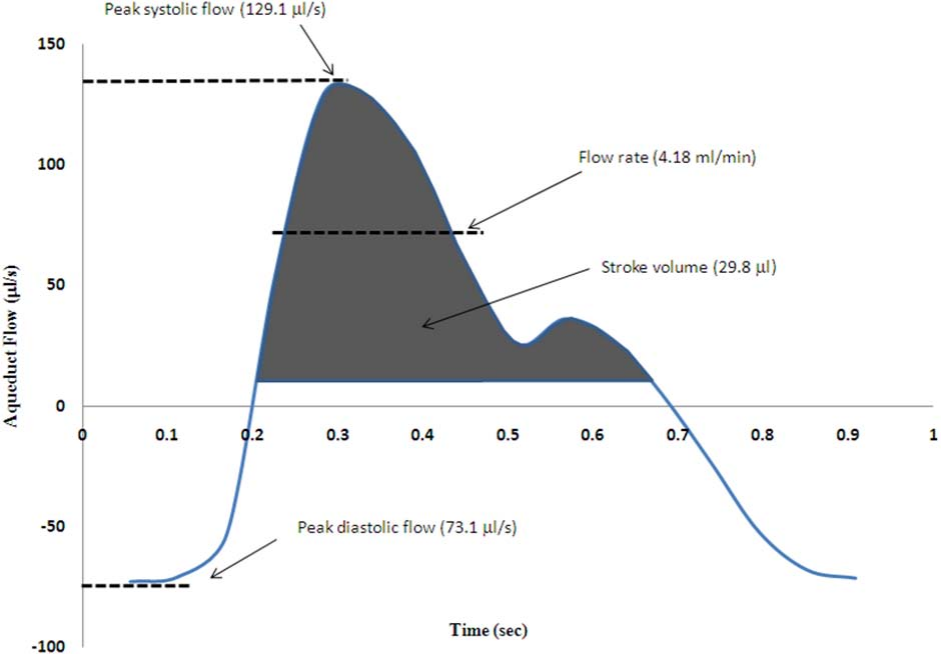
**Example of MRI flow waveforms in the cerebral aqueduct**. A typical MRI-derived flow waveform, demonstrating the possible measures extracted for quantification. Stroke volume is the most common parameter used, and is a measure of the net flow through the vessel/region of interest, in one direction (*i.e*., over approximately half the cardiac cycle). Flow rate has also been used frequently, and is the mean flow rate for flow in one direction. Peak flow is used less frequently, and is a measure of the highest (*i.e*., systolic, or lowest for diastolic) flow rate over the entire cardiac cycle.

Most MRI-derived amplitude measures are absolute measures such as stroke volume, flow rate, peak systolic flow, and peak diastolic flow (see Figure 6 for examples). Stroke volume, an appropriate outcome for flow but not for pressure measurements, is a gauge of the net volume of fluid pulsating back and forth over the cardiac cycle [50], and is typically reported in μl or ml. This is the predominate measure for CSF flow measurements [50-55], with the exception of cervical CSF flow in Chiari malformation studies where the anatomical pathology often creates localized jets of CSF flow and *peak *flow measures are more representative of the disease [56-58]. Flow rate, or mean flow, is the average flow in one direction (or for unidirectional flow, average flow above the mean) and is related to stroke volume, except that flow rate is affected by heart rate; to rough approximation, stroke volume and flow rate are related by stroke volume = flow rate/heart rate/2. While not as commonly used a measure as stroke volume, it has been used by various groups for diagnostic [59,60] and prognostic [61-64] applications in HC.

Peak systolic or diastolic flow (or velocity) can also be extracted from the flow (or velocity) waveform and has been used as another amplitude measure from MRI data, although examples are rare [65-67]. In particular, peak velocity measurements should be carefully scrutinized due to the potential for error. This concern arises mainly with respect to measurements in the aqueduct, where changes in measurement location can have a profound effect on peak velocity. Consistency of technique (*e.g*., positioning of the imaging slice) is crucial in such studies to ensure reliable results. A limited number of MRI phase contrast studies have reported results using PI [68,69], but in general this is not the preferred measure since PI includes effects of pulsatility and mean flow, both of which can be altered with disease.

### Other aspects of the pulse wave: pulse wave timing

**Figure 7:**
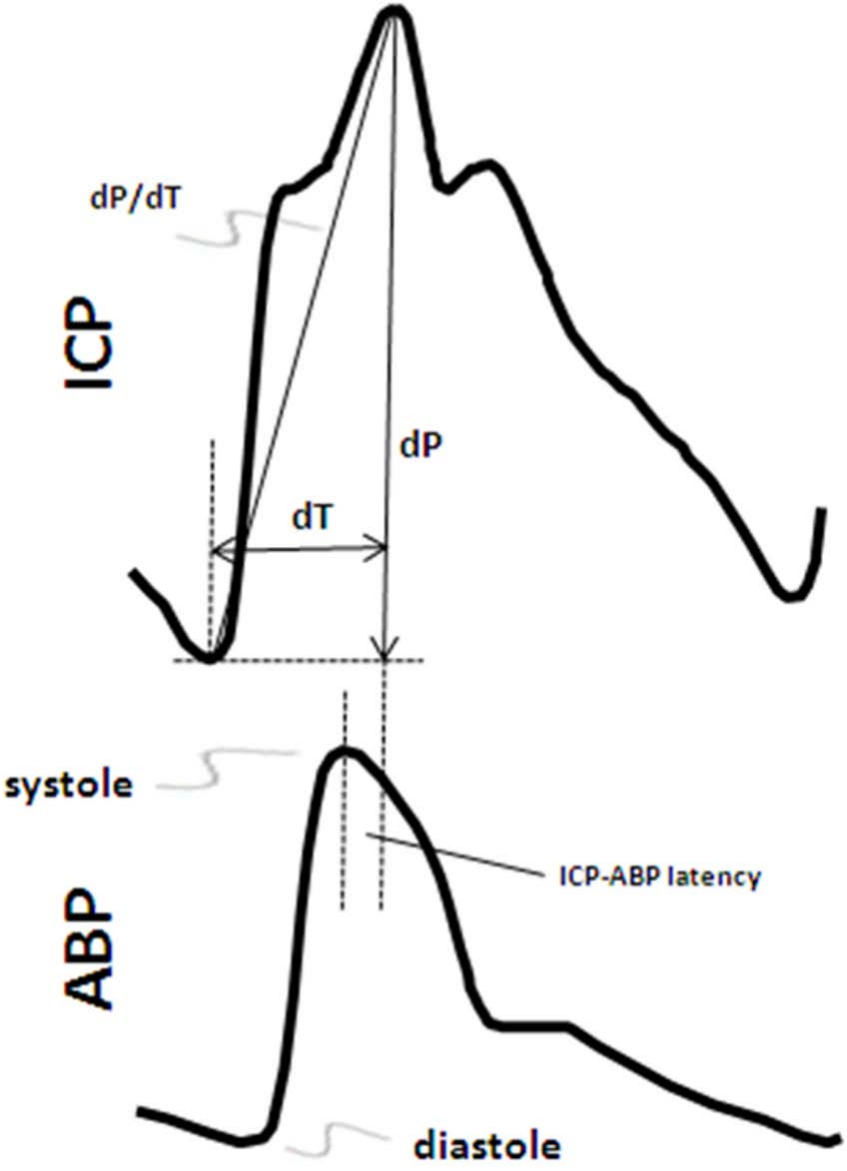
**Timing aspects of the intracranial and arterial pressure waveforms**. Single-pulse intracranial and arterial blood pressure waveforms, showing the elements of pulse wave timing. Timing can either be calculated intrinsically within the intracranial pulse wave, such as with the systolic/diastolic timing difference (dT) or the slope of intracranial pulse wave (dP/dT), or it can be measured relative to a reference pulse wave, such as the latency between the peaks of the ICP and the ABP waves.

A more subtle feature of the pulsatility waveform (and more difficult to extract) is its timing. Figure 7 illustrates the elements of pulse wave timing. The timing of a pressure or flow waveform in the cranium is most affected by intracranial compliance; a "loose" or more compliant cranium will transmit pulses more slowly than a "tight" or less compliant one. The most important consideration for collecting this information is that timing is relative, and a "reference" waveform is usually needed. For example, the pressure from an arterial line may be used as a reference for the ICP waveform. The arterial pulse waveform is the most appropriate reference, because it is the arterial blood flow which drives intracranial pulsations, but is also the most invasive and difficult to collect and has generally only been used in pre-clinical studies [70-74]. A non-invasive pressure reference can be obtained from the systemic blood pressure, but is considered less reliable (due to timing differences between brachial and carotid waveforms). Nonetheless, Piper *et al *used the non-invasive blood pressure waveform as a reference in both animal and patient TBI studies, and were able to show significant differences in timing between mild and severely impaired patients [35,75]. The electrocardiographic waveform can also serve as a reference [76]. In an attempt to bypass the need for a reference waveform, Eide used the latency between the diastolic and systolic peaks as a timing measure, but was unable to show differences between patients based on improvement following shunting [77] (even though a more recent study found measurable differences between groups when using the arterial blood pressure (ABP) waveform as a reference [78]). A vascular timing reference is more readily obtained in MRI studies where flow can be measured in both CSF and in intracranial arteries or veins [54,68,79-81]. Such studies have successfully shown timing differences between arterial and venous flow in hydrocephalic patients compared to age-matched controls [79,80].

Information on the relative timing between intracranial and reference waveforms can be extracted either in the time-domain or in the frequency-domain. In the time-domain, the peak-to-peak difference in timing is used, such as the arteriovenous delay [79]. The main difficulty with reliability of this information is that, while the two waveforms being compared are cardiac-driven, they are often measured differently (*e.g*., using different measurement techniques, or in different parts of the body) resulting in waveforms with different morphologies. This results in different degrees of waveform distortion and considerable variability in the peak locations, particularly when comparing different patient populations. On the other hand, this distortion may be of clinical relevance: Takizawa *et al *showed that the normal distortion of ICP waves is reduced during intracranial hypertension [82].

The frequency-domain approach effectively solves this distortion issue. The individual frequency components of the Fourier transformation each consists of a pure, distortion-free sine wave, so by looking only at individual components, distortion-free timing measurements are possible. For the cardiac-driven waveform of interest, the primary component is usually at the heart rate frequency, and the timing information (called "phase" in methods such as Fourier transformation and time-varying transfer function analysis) at this frequency can be compared between the primary and reference waveforms. This method has been used in both pre-clinical models [70,72,83] as well as in clinical investigations of TBI [34,35,84-86] and HC [81,86-88].

The parameters discussed above relate to the real-time variation of the pulsatile waveform within the cardiac cycle. However, there is another element of timing which can be considered in waveform analysis, that is, long-term variations in pulsatility over many minutes or hours. Such an assessment is only possible with ICP, since indwelling catheters can monitor pressure waves continuously. Interestingly, it has been found that even under pathological conditions pulsatility is not necessarily elevated all the time. Criteria for determining disease severity and prognosis, based on the percentage of time during which the pulse pressure amplitude is elevated, have led one group to develop a highly predictive model for guiding shunt surgery in HC [77].

### Other aspects of the pulse wave: Pulse wave shape

Intracranial pressure or flow waveforms have a unique morphology, and changes in the morphology have also been used as a clinical marker of disease. At normal ICP, the pressure waveform exhibits characteristic peaks and dips, mostly due to changes in pressure and flow with opening and closing of the myocardial valves. Pre-clinical [74,89] and clinical studies [90-92] have shown that these features are smoothed out and ultimately disappear with intracranial hypertension, a result similar to those of Takizawa *et al *noted above [82]. One group has developed intricate mathematical algorithms to detect changes in these peaks and dips, correlating them with changes in ventricular size in HC [93]. Utilizing this type of information, they were recently able to predict episodes of elevated ICP twenty minutes *before *they occurred [94].

### Systems analysis vs. raw data methods

All of the analyses described above are, for the most part, based on a raw data analysis philosophy. That is, they make the assumption that the pulse wave is *independently *related to intracranial physiology and changes in the pulse wave can be related to disease pathophysiology and to patient prognosis. However, these analyses do not make any assumptions about how this pulse wave is generated and how the arterial pulse responsible for intracranial pulsations might vary from patient to patient and, more importantly, how it might be altered in disease. In contrast, a systems analysis approach attempts to look at intracranial pulsations as part of a complete pulsatile system, and the main goal of the analysis is to describe how the system transforms the input (*e.g*., the arterial blood pressure waveform) into the output (*e.g*., the ICP waveform). The input and output are then collected over time. In engineering terms, these time-series are called *signals*, and a function which generates one signal from another is called a *system*. In our case, the real analogue for the system is the cranial cavity, vessels, brain, and CSF. With enough data, we can mathematically characterize how the system transforms one signal into another. This is called *systems identification*. An advantage of the systems approach is that formal mathematical approaches exist for evaluating changes in the system itself (as in the time-varying transfer function method [73]), as well as the extension to multiple inputs or multiple outputs of the system. By considering how the system changes with a disease process or a proposed treatment, quantitative understanding of an intervention becomes possible.

**Figure 8:**
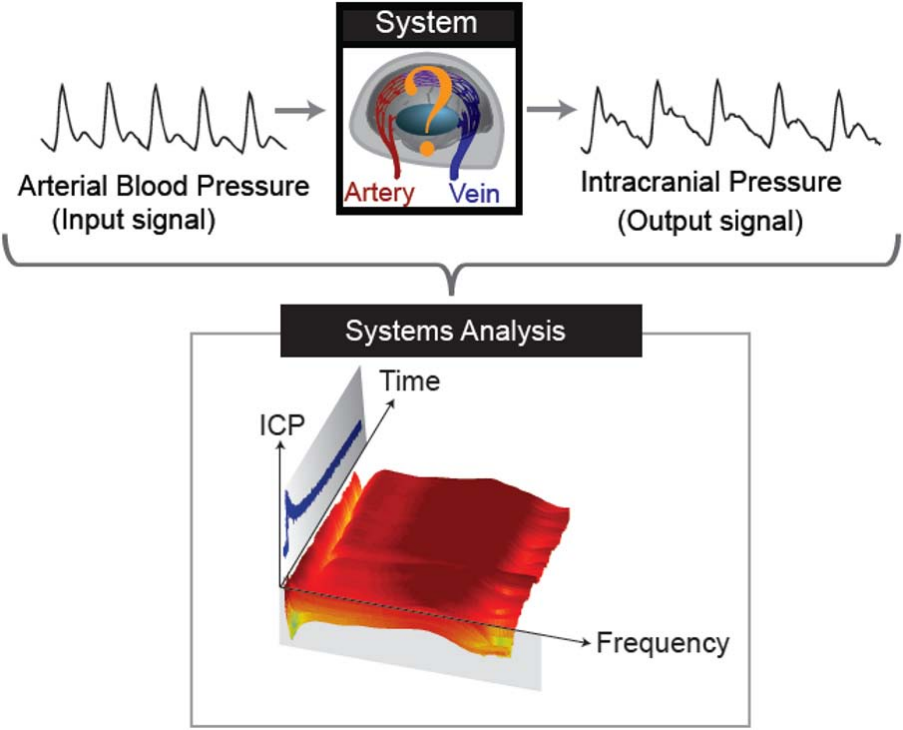
**Systems analysis of the intracranial pulse pressure and the concept of transfer function**. Because the intracranial pressure wave is a complex result of both the shape of the incoming arterial pressure wave, as well as the biomechanics of the intracranial compartment, additional analysis is needed to extract information about the biomechanics of the intracranial system independent of pressure waveform morphology. In systems analysis, the concept of transfer function is used to accomplish this. In these experiments, both arterial and intraparenchymal pressure were measured. The frequency-domain transfer function relates these two waveforms, *i.e*. how does the system (the cranium) transform the input (arterial pressure) into the output (parenchymal pressure)? This work showed the existence of a "notch" in the transfer function specifically in the vicinity of the heart rate (dip in signal seen in the lower right-hand corner) indicating minimal transmission of the fundamental cardiac frequency from the arterial pressure into the parenchymal pressure. However, under conditions of raised ICP through CSF volume loading, this notch disappears (reddish area just above the lower right corner, coincident with the increase in ICP seen in the blue curve) because of the increase in the fundamental cardiac frequency component of the intracranial pressure wave (figure reproduced with permission, with modifications, from Zou *et al *[73]).

As an example, systems analysis has been used to identify a pulsation absorber mechanism in the pulse wave response of the cranium to CSF volume loading in canines [73] (see Figure 8 for further explanation of the transfer function concept). The identification of an absorber mechanism specifically at the cardiac frequency indicates an important role for CSF pulsations in preventing strong arterial pulsations from entering the cranium and having potentially damaging effects on the cranial microvasculature. Similar methodology was recently implemented in a canine model of obstructive HC, showing the deterioration of the pulsation absorption mechanism in chronic HC [95]. These results highlight the importance of complex data analysis techniques with a systems approach in interpreting intracranial pulsatility measurements, and their changes with disease.

## The intracranial pulse wave - preclinical studies

The earliest investigations into intracranial pulse waves, their origin and their changes with disease, date back to the work of Bering in the 1950's [96,97] and later to Dunbar in the 1960's [98]. Most of this early work was performed in dogs, and led to the conclusion that the intracranial pulse wave is a product of the arterial pulsations entering the cranium, and is only influenced secondarily (*e.g*., in morphology) by venous pulsations. Hamer was one of the first to look at physiological modifications of the pulse pressure wave, also concluding that the arterial pulse wave predominately determines the pulse wave, except under conditions of cardiac insufficiency and increases in central venous pressure, when it can take on more venous character [99]. Interestingly, this work was one of the first to suggest that alterations in brain tissue compliance could have a deleterious effect on the pulse wave and might affect "vascular damping" of the arterial pulse wave with subsequent transmission of the pulse wave into the cerebral capillary bed.

Portnoy and Chopp continued this work throughout the 1980's, and were the first to use systems analysis of the ICP wave [29,30,70]. While their basic conclusion may have been similar to prior work, *i.e*. that the amplitude of the pulse wave generally increases with changes in physiology from normal conditions, the use of systems analysis allowed them to add the important finding that most of this increase occurs at the fundamental cardiac frequency. This observation was thus the first to show that there are frequency-dependent changes in the pulse pressure wave which may have relevance to normal brain function and its change with disease. Furthermore, in contrast to the earlier work, they concluded that the intracranial pulse wave is primarily venous in nature: the transfer function between the intracranial wave and the pulse wave in the sagittal sinus was close to 1, while the arterial/intracranial transfer function was much different from 1 (this conclusion is not generally accepted and the source of the intracranial pulse wave is assumed to be mostly arterial. According to this assumption, the similarity of the venous and intracranial pulse wave would then be due to the transfer of pressure waves from the parenchyma into the veins, rather than *vice-versa*). More recent work has further supported the importance of frequency-dependent changes showing that the unique response transfer function at the cardiac frequency is similar to a resonant notch filter, a response which may serve to prevent the primary component of the arterial pulse pressure wave from being transmitted into the intracranial pulse wave under normal conditions [73,74]. The frequency dependence of a difference in gain (how the input *amplitude *is translated to the output *amplitude*) or phase, suggests that the concept of a unique, single-value "compliance" which would relate any shape of input to the output (*i.e*., regardless of frequency) is an oversimplification. Systems analysis using transfer functions, however, allows consideration of a multi-value compliance as a function of input frequency--and the specific behavior of this function near the observed heart rate is of particular interest for probing the ability of the cranium (*i.e*., the system) to absorb the pulsatile energy due to the cardiac pulsations.

**Figure 9:**
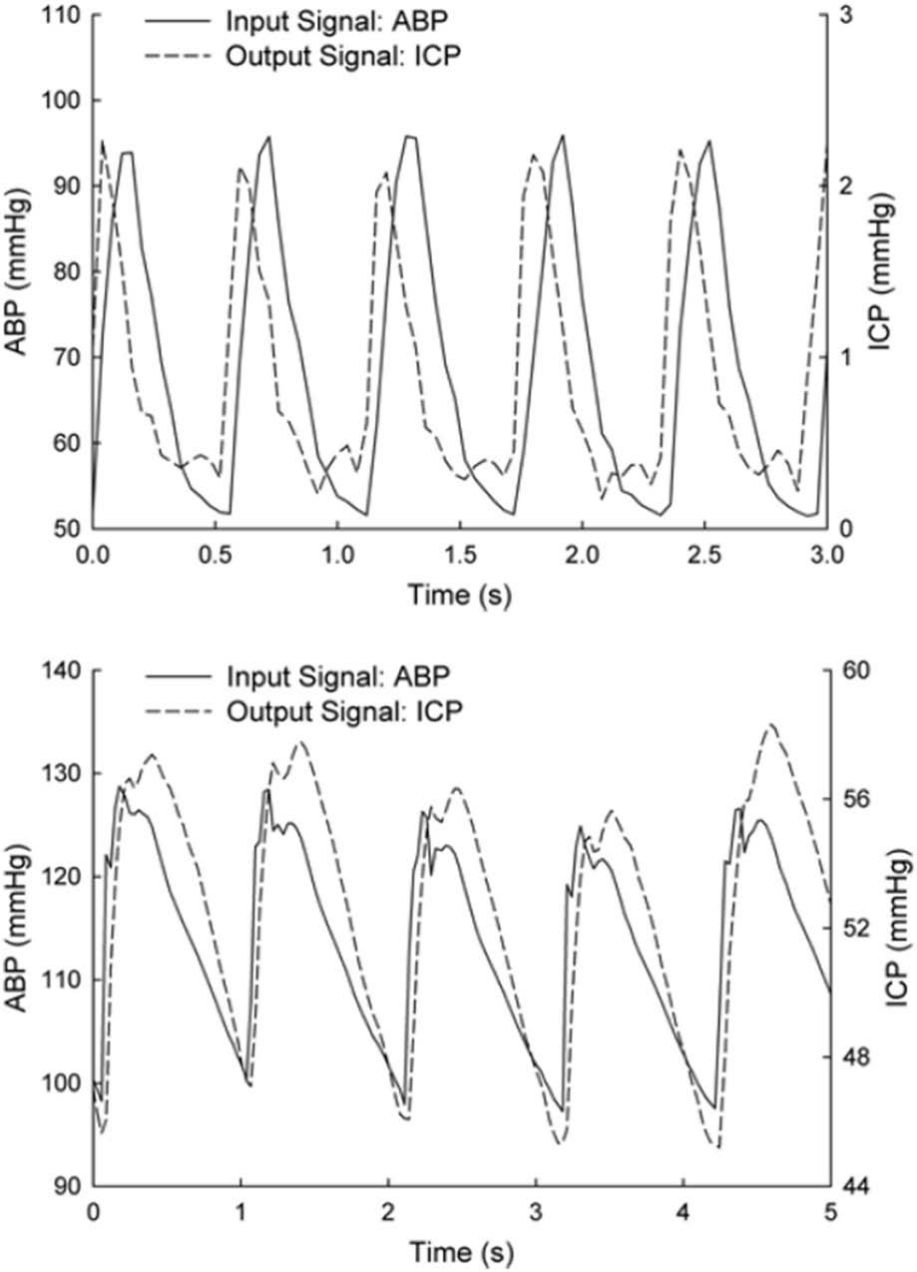
**Rounding of the intracranial pulse wave as a result of increased ICP**. Elevated ICP leads to decreased intracranial compliance, which investigators have found to result in amplification of the lower harmonic content of the intracranial pulse pressure wave, relative to the higher harmonic component. This behavior appears as a rounding of the pulse wave demonstrated here by CSF volume loading in the dog (upper panel: normal ICP levels, lower panel: raised ICP condition). The data also illustrate the timing, or phase, difference between the ABP and ICP waveforms, and the phase change with changes with mean ICP (figure reproduced with permission from Wagshul *et al *[74]).

Intracranial pulsatility has also been investigated in animal models attempting to mimic diseases of impaired intracranial compliance, such as intracranial hypertension (*e.g*., via CSF volume loading) and hypercapnia. Portnoy and Chopp showed that while conditions of hypercapnia, hypoxia and volume loading all produced increased pressure pulsatility (as measured by the arterial-to-CSF transfer function), the latter condition produced less of a change at any given mean ICP [29]. In addition, these conditions all produced a "rounding" of the pulse wave, similar to that noted by other authors [74,82,100] (in the frequency domain, this is consistent with increased pulsatility primarily in the fundamental cardiac component). This effect is illustrated in Figure 9 (data reproduced from [74]). Using extensive systems analysis of pulsatility of the ICP wave, Piper and colleagues also showed that intracranial and arterial hypertension as well as hypercapnia produce increases in the pulse wave, with most of the change again occurring at the fundamental frequency [75]. Intracranial compliance was dramatically reduced during intracranial hypertension, but only marginally with hypercapnia. Unique to this study was the added use of phase information; while their finding of a negative phase shift (*i.e*., delay of the pulse wave) with reduced compliance during volume loading is not surprising, the positive phase shift (*i.e*., an earlier pulse wave) seen post arterial hypertension is an unexpected finding and highlights the importance of considering both compliance *and *blood volume in models of intracranial dynamics.

In work more directly related to disease pathology, Di Rocco and colleagues showed that manipulation of the ventricular pulse pressure wave could lead to ventricular enlargement [101]. They mechanically enhanced the ventricular pulse wave with an intraventricular balloon in sheep, and showed that the size of the manipulated ventricle increased compared to the contralateral one. This was the first demonstration of the importance of CSF pulsatility with respect to ventricular dilation. Throughout the 1980's, investigators continued to show the importance of the CSF pulse wave, in particular in animal models of HC, mostly involving kaolin injection into the cisternum magnum [16,18,83,102-105]. Foltz and colleagues demonstrated a marked increase in resting state pulsatility as well as in the pulsatility response to increases in mean pressure [16]. Again using systems analysis methods, Portnoy and Chopp showed a marked increase in the amplitude of the pulse wave with HC induction, although there was no correlation with ventricle size [83]. By observing arterial (systemic pressure), CSF (ventricular) and venous (sagittal sinus) pressure waveforms, they were able to investigate the effect of both arterial and venous pulsatility on the CSF pulse wave. The primary conclusion was that pulse wave changes in HC are very similar to those due to intracranial hypertension and are not unique to HC.

More recently, Penn and colleagues used a dog model of HC to show that there is no transmantle gradient (*i.e*., difference in pressure between ventricle and cortical subarachnoid space), either in mean pressure or in the pulse pressure [106]. This result held both during the acute development phase of the disease, with markedly increased mean and pulsatile pressure, and in the chronic phase with normalized mean and pulsatile pressures. The existence of a transmantle gradient (either in mean or pulse pressure) has been hypothesized as one possible explanation for ventricular dilation in HC [107,108], although a recent study by Eide and Saehle in NPH patients showed no evidence of pulsatile trans- or intra-mantle pressure gradients [2].

All of the studies considered above used direct measurements of ICP and the pressure pulse. The advent of transcranial Doppler ultrasound allowed the non-invasive study of intracranial pulsatility in vascular flow. Before proceeding to review these studies, however, a word of caution is in order, as noted above. TCD studies look at flow in intracranial blood vessels, while invasive pressure measurements typically observe parenchymal or ventricular pressure. While ICP pulsatility and intravascular flow pulsatility are certainly related, they are measures of different aspects of pulsation in the brain and distinct differences can be expected. The number of studies investigating changes in TCD-based pulsatility in an animal model is quite limited, presumably due to the ease with which TCD can be done clinically and its non-invasive nature. Clinical studies will be reviewed in detail below. Nonetheless, as with pressure monitoring investigations, the few studies that exist generally found an increase in flow pulsatility with intracranial hypertension, again an indication of the reduced intracranial compliance [109-113]. Czosnyka *et al *used TCD in rabbits to observe changes in PI with intracranial hypertension, and concluded that PI can be a good indicator of cerebrovascular resistance, but only under conditions of intact perfusion pressure [110].

The relatively new technology of MRI has also only been applied to animal models in a few instances, likely because of the expense of MRI technology and its ready availability in clinical studies. Wagshul *et al *found markedly increased aqueductal CSF flow pulsatility in a rat model of HC [114], a result which has been well documented in communicating HC patients, and Alperin *et al *have shown elevated CSF flow pulsatility at the CCJ by volume loading [115] in a baboon model. A unique dog model of Chiari malformation, a condition in which jet-like pulsatile CSF flow occurs at the CCJ, has been used to document pulsatility changes with this condition [116].

In summary, numerous studies over the last three decades, mostly using invasive pressure monitoring, have led to the general conclusion that pressure pulsatility serves as a sensitive indicator of intracranial compliance, with the increase in pulsatility in HC being an indication of reduced intracranial compliance due to raised ICP and compression from the enlarged ventricles. Studies have also shown that there are important frequency-dependent factors which affect the way the pulse wave is transmitted into the cranium and how it is changed with disease. However, no study has clearly demonstrated the importance of intracranial pulsatility as a causative factor in the development of the disease process in either HC or TBI.

## The intracranial pulse wave - clinical studies

### Clinical applications: ICP

Intracranial pulsatility has been measured clinically for years, ever since the report of Bering in 1953 [97], and until the advent of transcranial Doppler, the only evidence of these pulsations was from invasive pressure monitoring. Foltz reported that the intracranial pulse pressure was 2-3 times higher than normal in communicating HC, with "an even more striking pulse pressure increase" in obstructive HC cases [16]. They also noted that the peak of the pulse wave occurred earlier than normal in these patients; in our view, another indication of the reduced intracranial compliance. Avezzat and colleagues showed a marked increase in pulse pressure in a various etiologies (*e.g*., HC, brain tumors and intracranial hypertension), and demonstrated a linear relationship between increased pulse pressure and the pressure-volume response, yet another clear indication of altered intracranial compliance [17]. However, they also noted a word of caution in using such pulse pressure as a reliable measure of intracranial compliance: changes in pulse pressure also depend on changes in cerebral blood volume which are usually unknown. There are, however, some studies which contradict these general conclusions of elevated pulse pressure in HC. Matsumoto *et al*, for example, "could not find high pulse pressure of the ICP pulse wave" in their communicating HC patients [18].

It may be that some of the variability in results is related to the period of time over which ICP and pulse pressure are monitored. For example, it was recently shown that the relationship between mean ICP and pulse amplitude is dynamic and non-linear; the expected finding of high pulse amplitude when mean ICP is high and *vice versa *was only seen 60% of the time [117]. Moreover, shunt-responsive NPH patients (with so-called "normal" pressure) nonetheless have elevated pulse pressure, with amplitudes comparably high to those in stroke patients in the intensive care unit [118]. These observations may indicate that it is the reduction of intracranial compliance, and not necessarily raised ICP, which causes the elevated pulse pressure amplitudes.

**Figure 10:**
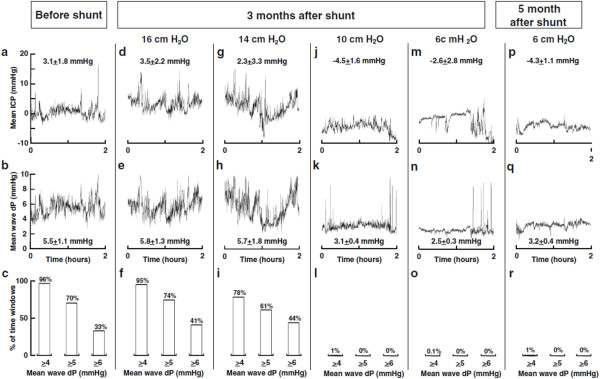
**The effect of shunting on mean pulse wave amplitude**. Mean amplitude of the pulse pressure wave has been used both as an indication of disease severity and as an indicator of the likelihood of shunt success in hydrocephalus. In this patient, it can be seen that not only is the mean wave amplitude dramatically reduced following shunting (leftmost vs. rightmost column, middle row), but that it is also a sensitive means of adjusting the shunt valve opening pressure (four central columns, middle row, figure reproduced with permission from Eide and Sorteberg [25]).

To quantify these temporal effects, Eide and colleagues developed a method for analyzing single wave pulsatility in the time domain [119]. While the pulse pressure measured with this technique is very similar to that used in other studies, the authors used a very different philosophy in analyzing their data. Long-term monitoring was used, typically 6-12 hours, and the pulse pressure then categorized based on the percentage of time it remained above a certain critical value. For example, in one study there was a clear demarcation between patients who improved following shunting compared to those who did not, based on the percentage of time windows for which pulse pressure was above 4 mmHg (85% for improved vs. 45% for unimproved) [22]. In another study, pulse pressures before and after shunting were clearly differentiated based on the percentage of time above 4 mmHg (80% before vs. 30% after) [25] (see Figure 10). This technique highlights the dynamic nature of the pulse pressure, showing that even under pathological conditions the pulse pressure is not necessary always high.

One major advantage of invasive monitoring as compared to non-invasive techniques is the ability to *simultaneously *monitor the pulse wave in different intracranial regions, providing the opportunity of comparing pulse pressure in different compartments. While the mean ICP varies between locations, because of differences in baseline pressure (*e.g*., related to sensor calibration) and hydrostatic pressure gradients, cardiac-induced pulsatility would appear to be independent of sensor location [1,2,120] (although there are differences between cranial and spinal spaces, with amplitudes about 2 mmHg lower in the lumbar space [121]). A recent study, however, showed that some HC cases are associated with pulse pressure gradients, and these gradients may be related to the disease process [122]. More studies are needed to confirm this important finding.

**Figure 11:**
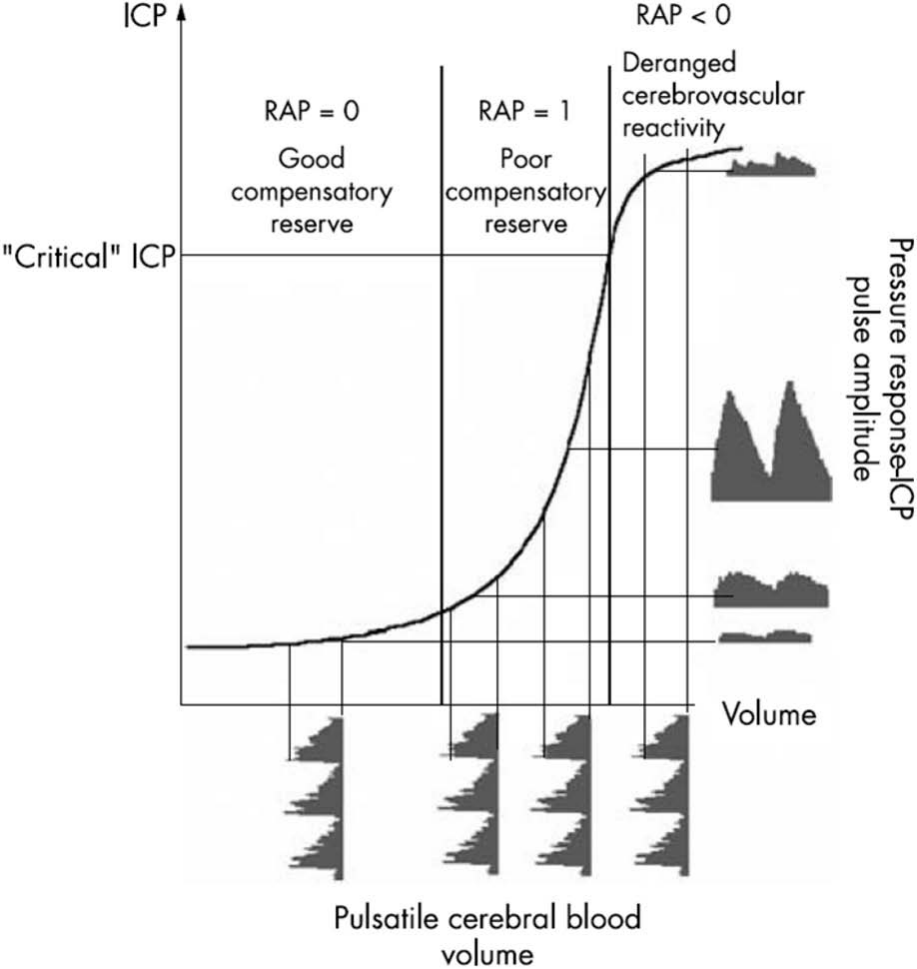
**Correlation between pressure and pulse amplitude (RAP)**. The RAP concept can best be understood through this figure showing the expected pulse amplitude behavior with increasing ICP. Under normal ICP conditions (left), the shallow slope of the pressure-volume curve leads to a weak relationship between pulse amplitude and pressure; RAP is close to zero. As ICP rises (middle), and with it the slope of the pressure-volume response, there is a clear *positive *correlation between pulse amplitude and mean pressure; as pressure rises, so does pulse pressure, resulting in an RAP close to 1. This relationship indicates a loss of compensatory reserve in the pressure-volume response. Finally, when ICP reaches a critical point (right), the slope of the pressure-volume curve decreases sharply resulting in a *negative *pulse amplitude-pressure relationship; RAP becomes negative. In TBI, negative RAP has been shown to predict patients who are unlikely to recover (figure reproduced with permission from Czosnyka and Pickard [200]).

Because of the obvious significant risk of marked elevation of ICP following TBI, this is another field which has seen increased interest in using pulse pressure waveforms for clinical diagnosis. While ICP monitoring has been used for decades in this population, it was not until the mid 1980's that investigators began to utilize the pulse pressure for diagnostic purposes. Czosnyka *et al *used frequency domain analyses and introduced the concept of AMP or amplitude of the fundamental frequency component, and showed a close correlation with ICP [31]. Interestingly, they also noted that by using only the fundamental harmonic component of the pulse wave for their calculation of AMP, as opposed to the peak-to-peak amplitude of the waveform, a much better correlation to mean ICP was obtained. In this work, they also introduced the concept of pulse pressure variability (denoted RAP) as a measure of compensatory reserve of the craniospinal compliance, and showed that this measure can be used to distinguish patients who will recover from those who will not [84]. Figure 11 illustrates the elements of the RAP technique. One disadvantage to this approach is the use of lumbar infusion, which the authors argue is necessary in order to manipulate the ICP in a controlled manner, as compared to most other work which relies on observation of the natural course of the intracranial pulse pressure. Nonetheless, this work has shown important results; more recent studies with these techniques have explored the potential benefits of decompressive craniectomy and its effect on ICP dynamics [123]. These techniques have also been applied to HC patients, and can be used to distinguish ventricular dilation in HC from brain atrophy, although there is some overlap between these populations [88].

Systems analysis of the pulse pressure waveform has also been used in TBI [34,35,75,124,125]. As compared to other studies, however, this work has focused not on the fundamental cardiac frequency, but on the higher harmonic components. Following up on earlier work with volume loading in dogs [72], it was shown that there exists a high frequency resonance which is a natural characteristic of the intracranial cavity and highly dependent on intracranial compliance. The systems analysis approach can be a very powerful tool in that different portions of the frequency spectrum may be indicative of various aspects of the pathophysiology. For example, Piper *et al *showed that TBI patients could be categorized into four different characteristic frequency-domain patterns, which they associate with changes in cerebrovascular tone (low frequency region) and intracranial compliance (high frequency region) [35]. Lin *et al *similarly used systems analysis to show the existence of a high frequency component which was only present in TBI patients with good outcome. This feature disappeared in patients with moderate or poor outcome, which the authors interpret as a pathological increase in cerebrovascular resistance, not as a change in intracranial compliance as is usually assumed [125]. This work also highlights the advantage of using systems analysis over more straightforward waveform analysis; only the systems analysis approach was able to differentiate patients with good from those with intermediate outcome. Unfortunately, these techniques have never developed into a viable tool for predicting TBI outcome, possibly due to the technical complexity and the high variability of results with changes in heart rate [126].

Studies beginning in the late 1990's began to attempt to utilize invasive pulse pressure monitoring for guiding HC therapy. As with the studies noted above, all showed increased pulse pressure with disease, but there has been much disagreement as to whether or not this increase can be correlated with successful therapy. In idiopathic NPH patients, Barcena *et al *showed that the pattern of increased pulse pressure was well correlated with decreased pulse wave latency (both an indication of decreased intracranial compliance), and that this pattern was clearly distinct from the pattern seen in healthy subjects as well as in cases of brain atrophy, where increased pulse pressure was correlated with increased latency [20]. However, within the shunted group, they were unable to differentiate improved and unimproved patients based on either amplitude or latency of the pulse wave; one other study found similar results [127].

**Figure 12:**
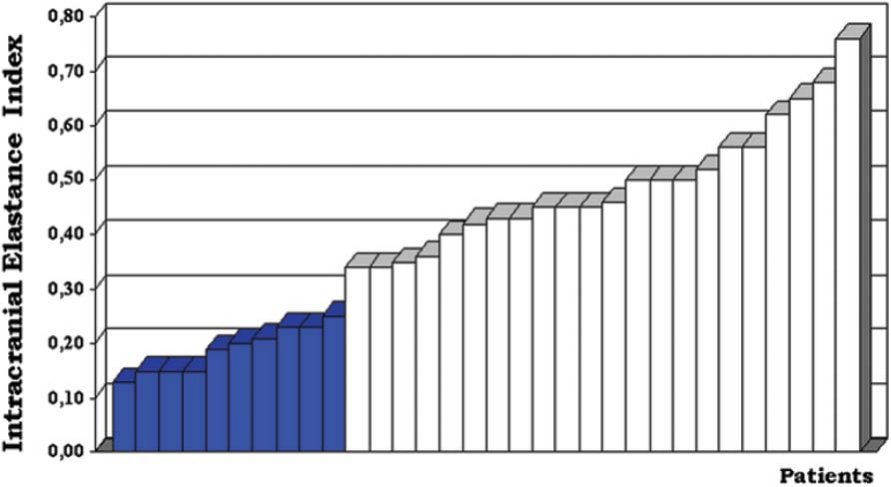
**The importance of intracranial compliance in hydrocephalus management**. In this work, intraventricular infusion tests were used to measure the slope of the pressure-volume curve. From this, the authors derive an intracranial elastance index - not the absolute elastance because they use diastolic pressures rather than mean pressure in the calculations - which is shown here to provide excellent separation between patients who improved (white) and those who did not improve (blue) following shunting. The elastance index used here is proportional to the inverse of intracranial compliance (figure reproduced with permission from Anile *et al *[129]).

On the other hand, a number of recent studies have shown promise. Brean and Eide showed pre-surgical pulse pressure to be "highly related" to shunt response, although they had a 16% false negative rate [24]. In another study by the same group of 130 idiopathic NPH patients, clinical response to shunting could be anticipated in 93% of patients with elevated pulsatile ICP (determined by mean pulse amplitude ≥4 mmHg on average over-night and >5 mmHg in ≥10% of observations), while only 10% of patients with low pulse amplitude improved [128]. A very recent study showed excellent separation of responders and non-responders using an intracranial elastance index [129], derived from the slope of pulse pressure vs. mean ICP curve during intraventricular infusion (elastance is the inverse of compliance, see Figure 12). Using a systems analysis approach, Eide *et al *were able to separate responders from non-responders based on both pulse amplitude and phase information (relative timing difference between the ICP and ABP pulse waves, which was smaller in responders) [78].

In summary, recent advances using quantitative measures extracted from the pulse pressure waveform have shown very promising results and all would appear to support the view that intracranial compliance and its effect on the intracranial pulse amplitude can play a critical role in HC and TBI management.

### Clinical applications: TCD

TCD flow velocity measurements follow the same trend as pre-clinical studies discussed above, most indicating an increase in PI with pathology [130-141], and good correlation with clinical condition [139,142]. Using a technique similar to that of Eide described above, Splavski *et al *showed good correlation between the degree of elevated PI and the duration of time (in hours per day) for which the mean ICP was elevated (ICP > 25 mmHg) [138]. Others have used more straightforward PI measurement and found good correlation with raised ICP [131,143,144]. One study, however, found very weak correlations and concluded that the technique was not adequately sensitive [132].

With respect to prognosis, PI has been found to fall following various surgical interventions, such as shunting [131,132,135,137,140], CSF drainage [145] and endoscopic third ventriculostomy [134] in HC and surgical decompression for TBI [146,147]. However, the same word of caution noted above is needed when considering these studies. While increased PI is often regarded as a measure of reduced intracranial compliance (*i.e*., a shift from the normal pressure-volume curve), because PI is a ratio of absolute pulsatility to mean flow, in many of these cases the increase in PI may result from decreased cerebral blood flow rather than to an increase in absolute pulsatility. In our view, this may explain the wide variability in a recent study of PI following shunting in HC [137]. Nonetheless, these authors concluded that TCD may be a valuable tool when used in conjunction with other clinical information.

### Clinical applications: MRI

Clinical MRI studies of flow pulsatility have mainly focused on measurements in the cerebral aqueduct because of the high flow velocities, although there are a limited number of studies of flow in the prepontine cistern [51], at the CCJ [53,148], and in the cervical and intracranial vasculature [79,81,115,149-151]. The very early MRI evidence of pulsatile flow in the aqueduct was actually not obtained through quantitative measures, but deduced from a flow artifact leading to decreased CSF signal which is accentuated with increased flow velocities [152-155]. The application of the phase-contrast technique to quantify pulsatile CSF flow was developed in the early 1990's. Greitz [156] and Naidich *et al *[49] extensively documented CSF flow and brain motion using phase contrast MRI. These studies demonstrated the ability to quantify CSF flow through the cerebral aqueduct, in the prepontine cistern and at the craniocervical junction, as well as to identify patterns of brain motion. Based on these studies, it was concluded that pulsatility results in a funnel-like motion of the brain, as if the brain were being pulled in systole by the spinal cord. This motion was interpreted as due to the venting of the brain and CSF through the tentorial notch and foramen magnum during the systolic arterial expansion [156].

**Figure 13:**
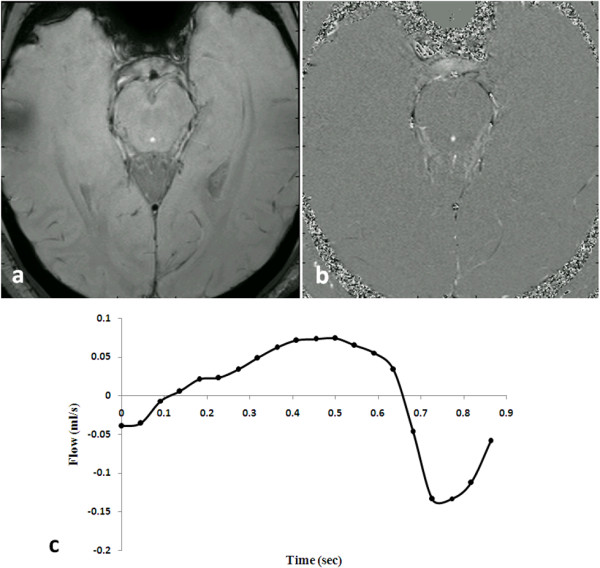
**Flow in the cerebral aqueduct from cine phase contrast MRI in a healthy control**. A typical MRI flow study, using the phase contrast technique, consists of the magnitude (a, anatomical) and phase (b, flow) image. In this example, cine images were taken as a function of the cardiac cycle by gating the image acquisition to a peripheral pulse signal. The image on the right depicts flow velocities during one phase of the cardiac cycle with caudal CSF flow (the bright dot in center of the image shows caudal flow in the aqueduct during this phase of the cycle). By summing over all pixels in the aqueduct, a net flow waveform is obtained (c). Stroke volume in this instance was 25.7 μl.

These landmark studies were followed by measurements of CSF flow in healthy controls, undertaken by numerous groups and focusing mostly on aqueductal flow and demonstrating reliable measurements [44,45,49,51,156,157]. Normal flow values (*i.e*., stroke volume) from these studies range from 30 - 50 μl [52,53,150,156]. A typical flow image and waveform is illustrated in Figure 13. Studies of CSF flow in the subarachnoid spaces have been less common, and have concluded that pulsatile CSF flow through the aqueduct is, in healthy individuals, but a small fraction of net CSF flow pulsatility, with normal aqueduct-to-CCJ flow ratios ranging from 4 [158] to 11% [148]. MRI flow measurements in the prepontine cistern can be used to characterize the ratio of supra- to infra-tentorial flow pulsatility, by quantifying the prepontine-to-CCJ flow ratio, and range from 25 [51] to 35% [48,158]. MRI studies in healthy controls have also documented the normal temporal relationship between arterial or venous pulsatile flow and CSF pulsations. In general, CSF flow in the inferior subarachnoid spaces is synchronous with arterial flow, while flow in the cerebral aqueduct lags by 15% of the cardiac cycle (*i.e*., 100-150 ms) [49,53,148].

MRI studies of flow pulsatility in disease have been primarily in HC, spurred on by the well-known changes in pressure pulsatility demonstrated with invasive monitoring methods discussed above. The primary finding is a marked increase in pulsatile aqueductal flow [50,52,59,61,62,67,156,159-167] with pathological values often rising as much as ten times normal. Luetmer *et al*, for example, used this measure to set a diagnostic criterion for separating idiopathic NPH patients from healthy controls (*i.e*., based on pulsatile flow rates either above or below 18 ml/min) [59]. Greitz *et al *reported a corresponding decrease in pulsatile flow through the CCJ [51], but these findings were from a limited number of patients, and one later study found no such change in a group of 12 communicating HC patients [148]. Aside from the amplitude of flow pulsatility, some investigators have also looked at temporal parameters as an indication of pathological dynamics. For example, Baledent *et al *have shown a shorter systolic flow period compared to healthy controls [148] and Miyati and coworkers have used systems analysis to show a highly significant correlation between the phase of the aqueductal pulse wave and pressure-volume response [162] (*i.e*., a linear relationship between timing of the CSF pulse wave and intracranial compliance).

MRI measurement of flow pulsatility at the craniocervical junction has been studied extensively in Chiari malformation. These studies have found increased heterogeneity in the flow pattern, consisting of both local flow jets and bi-direction flow [168-172]. The occurrence of flow jets necessitates the use of peak velocity, rather than net stroke volume, as the best indicator of pathology. Pinna and colleagues [173] used the temporal information from the flow waveform and found a shorter systolic CSF pulse in the ventral subarachnoid space of Chiari patients without a syrinx compared to those who had developed a syrinx (as well as compared to controls). In light of the discussions throughout the paper of the relationship between CSF pulse wave timing and compliance, these results would appear to indicate the important role of the intraspinal compliance and pulse pressure gradients in Chiari and the formation of spinal syrinxes [174]. In patients with syringomyelia in the absence of an obvious cause (such as Chiari or tumor), Mauer *et al *used phase contrast MRI to document blockage of CSF flow in the subarachnoid space surrounding the syrinx, finding this technique to be more sensitive compared to myelography [175]. Following surgical decompression, peak velocities decrease and flow waveforms change from "heterogeneous" to sinusoidal [56,171,176,177]. Alperin and colleagues used systems analysis to evaluate changes in intracranial compliance in Chiari, concluding that there was abnormal dynamics of the intracranial volume change over the cardiac cycle, which returned to "more normal-appearing dynamics" following decompression [178].

In comparison to these studies, which focus almost exclusively on changes in CSF flow pulsatility, Bateman and colleagues have studied changes in vascular flow pulsatility as a measure of flow pathology in HC [52,68,69,79,151], finding a significant *decrease *in the arterial pulse wave in NPH patients compared to age-matched controls. The change in arterial pulsatility, coupled with a marked increase in the aqueductal CSF pulse, led to a nearly two-fold decrease in the compliance ratio, a relative measure of intracranial compliance (the ratio of aqueduct to arterial pulse wave stroke volume) [52]. They have also shown changes in venous flow pulsation which may be an indication of the importance of venous pathology in HC [69,79]. Most significantly, they found decreased cortical vein flow pulsatility in patients, which reversed and surpassed control values following ventricular shunting. These studies also showed that vascular flow timing might be used as an indication of intracranial compliance changes in HC, with a marked drop in the arterial-venous delay (*i.e*., the delay between the arterial and venous systolic peaks) in patients, which reverses with shunting [79]. Unfortunately, at the end of the day, a study of shunt responsiveness concluded that none of the measured pulsatile flow parameters could reliably separate shunt surgery responders from non-responders [151].

**Figure 14:**
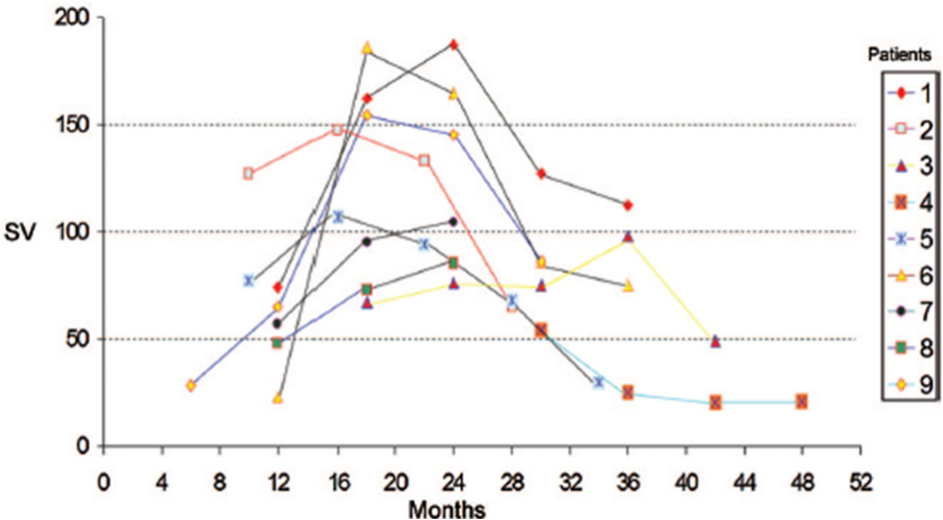
**Temporal changes in aqueductal stroke volume in unshunted HC patients**. Evidence that CSF flow can change over time with untreated disease may explain the difficulty clinicians have had using this measure for predicting shunt outcome. In this study, nine patients who had refused a shunt were followed over the course of four years (the time axis has been normalized for each patient, so that 0 months corresponds to the time of the first reported symptoms). The time at which MRI measurements are taken may play a critical role in their prognostic use for predicting shunt outcome. Normal stroke volume may only be indicative of poor shunt-responsiveness if taken at later time when stroke volume has decreased, perhaps due to irreversible atrophic changes in the brain which cannot be remedied with shunting. Normal stroke volume during the early development stages of the disease, on the other hand, may simply be an indication that intracranial compliance has not yet changed sufficiently to affect aqueductal flow patterns, and shunting may still prove effective in this patient group (figure reproduced with permission from Scollato *et al *[165]).

With respect to prognostic MRI studies, numerous studies have investigated the association between aqueductal pulsatility and outcome from shunting [50,54,61,62,67,159,161,163,167,179,180]. The first example of a prognostic, MRI-based flow pulsatility measure is the stroke volume measure; Bradley *et al *indicated favorable outcome for patients with aqueductal stroke volumes above 42 μl [50]. Other trials, mostly involving NPH patients, however, have not been promising. Using the same measure of stroke volume, but stratifying patients into low, medium and high stroke volume groups, Kahlon *et al *could find no statistically significant improvement in either cognitive or motor function in any of the pulsatility groups [163]. In another study using mean aqueductal flow rate, Dixon *et al *also found no significant association between CSF pulsatility and improvements in gait, cognition or urinary continence [62]. This same conclusion has been reached in a number of other recent trials [67,166,180]. In our view, scrutiny of these studies indicate that highly elevated flow pulsatility is usually a very good predictor of favorable outcome, but patients with normal or mildly elevated pulsatile flow levels will often also improve with shunting, leading to high false negative rates. Of course, some of the variability in results may be related to the temporal variability in pulsatility noted above from pressure monitoring studies [22,23,25,77], highlighting a distinct disadvantage of the MRI technique; because of the expense, only one point in a dynamically changing pulsatile system is captured. One unique recent study, in which aqueductal pulsatility was followed over a two year period in patients who refused a shunt, in our view may shed some light on this controversy. Scollato and colleagues showed that pulsatility can change over time with the development and progression of the untreated HC [165]. Aqueductal stroke volume was found to increase over a period of 1-2 years, but then to decrease over a similar timeframe (see Figure 14). Thus, it is also possible that this long-term variability in pulsatility, the source of which is still unknown, is one of the deterrents to accurately predicting shunt outcome using this particular measure.

An alternative treatment for HC, primarily reserved for obstructive cases, is endoscopic third ventriculostomy (ETV). Cine phase-contrast imaging is an important imaging modality for these cases; pulsatile flow through the stoma is used postoperatively to verify patency [181,182]. A number of publications have surfaced in the last few years, however, suggesting that ETV may also be an appropriate treatment in certain communicating cases. Greitz recently presented a hydrodynamic theory of communicating HC, arguing that ETV may be an appropriate therapy for restoring pulsatile dynamics without shunting [183]. Unfortunately, there are only a limited number of case studies which have looked at CSF flow (other than for stoma patency) before or after ETV. One study, in which most patients had elevated aqueductal stroke volume, showed only a small, non-significant decrease in flow pulsatility after ETV [184]. A more recent study found no association between ETV success and CSF flow pulsatility in the basal cisterns or at the cervicomedullary junction [185]. Thus, at present there is no obvious connection between CSF pulsatility and the success of ETV surgery.

In summary, the MRI techniques developed within the last twenty years have proved invaluable for non-invasive assessment of intracranial pulsatility in HC. Studies have consistently shown that HC is associated with elevated aqueductal flow pulsatility, as well as with changes in pulsatility in other areas of CSF and vascular flow. However, the strict association between pulsatile aqueductal flow and outcome from shunting remains an open question. A distinct, and we might even say likely, possibility is that flow pulsatility represents only a portion of the pathophysiology of the disease and additional non-invasive measures will need to be combined with flow measurements in order to adequately predict shunt responsiveness.

## Future directions

With respect to future directions in pulsatility research and its potential clinical use for both diagnosis and prediction of outcome, we would suggest that large-scale clinical trials are needed, with particular attention paid to uniformity in the definition of pulsatility measures to be collected and acquisition methods to be used. In particular with respect to MRI-based measures of pulsatility, the lingering disagreement in the scientific community about its usefulness appears to stem from the lack of consensus on imaging parameters/methods and data analysis techniques. Given the success of invasive pulsatility measurements in clinical prognosis [77,128,186], studies which can provide a link between changes in pulse pressure and changes in non-invasive TCD- or MRI-based measures of pulsatility will be particularly valuable. A careful consideration of pulsatile dynamics may make possible a clearer definition of when HC is adequately treated, which will in turn yield new ways to compare the mechanism of action of shunts, endoscopic fenestrations, and other therapeutic options such as pharmacological or genetic interventions, in the future. Consideration of the mechanisms of how pulsations are generated and received by brain and neurovascular tissue may also help us understand and ultimately guide therapy in headache or other mechanisms which resemble those encountered in HC.

Given the importance of intracranial compliance in conditions such as hydrocephalus and traumatic brain injury, which we have shown is central to the existence and changes in brain pulsatility, the ability to directly measure compliance may also play an important role in clinical decision making. Direct measurement of intracranial compliance, however, is technically difficult and usually invasive. Recently, Alperin *et al *have devised noninvasive methods for inferring intracranial compliance using MRI, based on the relative distribution of arterial, venous and CSF pulsatility at the craniocervical junction [115,187]. Such techniques may be the answer for a noninvasive method of assessing compliance changes with disease. For example, this technique has recently been used to demonstrate reduced intracranial compliance in NPH [188-190].

The studies we have discussed only imply a *passive *role for intracranial pulsatility, as an indicator of changes in brain compliance. More intriguing is the possibility that intracranial pulsations may play an *active *role in intracranial fluid dynamics, a hypothesis which has been suggested by a number of investigators [73,74,95,183,191,192]. By such a hypothesis, changes in the transfer of arterial pulsations into the surrounding subarachnoid spaces (*e.g*., in the basal cisterns) can lead to a redistribution of intracranial pulsations, with potential pathological implications if increased pulsatility redistributes to the capillary microvasculature. Indeed, a number of studies have documented decreased capillary density and caliper in experimental HC [193-196], and recent studies have shown that excessive *pulsatile *stress forces can change endothelial cell homeostasis and thus impair capillary hemodynamics through the potent vasodilator nitric oxide [197,198]. Whether or not such alterations at the microvascular level are a result of, as opposed to a cause of, the HC is still an open question, and is an ongoing investigation in one of our labs (MEW, unpublished results). The concept that adequate intracranial management of the pulsatile energy of the arterial input by free movement of CSF has been likened to the need for balance in net production and absorption of the fluid itself, even as far as referring to the need for absorbing pulsations as a "fourth circulation" in the intracranial compartment, in homage to the description of the CSF flow as the "third circulation" [199].

## Conclusions

The fact that everything within the cranial cavity pulsates with cardiac periodicity has been well established and studied over the last fifty years. While there have been numerous investigations of intracranial pulsatility, focused both on understanding these pulsations as well as on their relationship to neurological disease, these have not yet had a major impact on our approach to clinical diagnosis or treatment. We have shown a clear link between intracranial pulsatility and the compliance of the brain. This link certainly implies an important diagnostic role for intracranial pulsatility in diseases involving dramatic changes in the distribution of the intracranial contents; hence its importance in TBI and HC. While the search for noninvasive, prognostic tests utilizing pulsatility information is still underway, invasive monitoring of pulsatility is already being used at a number of centers and demonstrating its reliable, prognostic potential. Basic and clinical studies using noninvasive techniques have suggested correlations of pulsatile parameters with outcome, but the critical question is whether management decisions, which could not be made with already available time-independent measures, can be made on the basis of such analysis.

We have gained a tremendous amount of knowledge in the last six decades of research into the origins and significance of intracranial pulsatility in neurological disease. On the other hand, we are still in the early stages of the development of clinically useful techniques based on pulsatility-related measures. The validation of well-accepted modalities for improving patient outcome, using invasive and non-invasive modalities, as well as the formulation and testing of hypotheses regarding the many interesting pathophysiological questions, will depend on future technical advances in how we measure and analyze pulsatility, and our collective investigative imagination in broadening this field of research.

## Abbreviations

ABP: arterial blood pressure; CSF: cerebrospinal fluid; CCJ: craniocervical junction; ETV: endoscopic third ventriculostomy; HC: hydrocephalus; ICP: intracranial pressure; MRI: magnetic resonance imaging; NPH: normal pressure hydrocephalus; PI: pulsatility index; RI: resistive index; TCD: transcranial Doppler; TBI: traumatic brain injury

## Competing interests

MEW and JRM have no competing interests. PKE has partial ownership in dPCom AS, Oslo, which manufactures Sensometrics software used for ICP analysis.

## Authors' contributions

All of the authors contributed to the conception of the review in terms of overall content and focus. MEW contributed the bulk of the drafting of the article, while PKE and JRM contributed with thorough editing of the manuscript, and contributed data used for the figures. All authors have read and approved the final version of the paper.
